# Non-conventional induction strategies for production of subunit swine erysipelas vaccine antigen in r*E*. *coli* fed-batch cultures

**DOI:** 10.1186/2193-1801-2-322

**Published:** 2013-07-17

**Authors:** Adilson José da Silva, Antônio Carlos Luperni Horta, Ana Maria Velez, Mônica Rosas C Iemma, Cíntia Regina Sargo, Raquel LC Giordano, Maria Teresa M Novo, Roberto C Giordano, Teresa Cristina Zangirolami

**Affiliations:** Chemical Engineering Department, Federal University of São Carlos, Rodovia Washington Luís, km 235, São Carlos, SP Brazil; Genetics and Evolution Department, Federal University of São Carlos, Rodovia Washington Luís, km 235, São Carlos, SP Brazil

**Keywords:** SpaA antigen, *Erysipelothrix rhusiopathiae*, Bioreactor cultivation, Auto-induction, Lactose feeding

## Abstract

In spite of the large number of reports on fed-batch cultivation of *E*. *coli*, alternative cultivation/induction strategies remain to be more deeply exploited. Among these strategies, it could be mentioned the use of complex media with combination of different carbon sources, novel induction procedures and feed flow rate control matching the actual cell growth rate. Here, four different carbon source combinations (glucose, glycerol, glucose + glycerol and auto-induction) in batch media formulation were compared. A balanced combination of glucose and glycerol in a complex medium formulation led to: fast growth in the batch-phase; reduced plasmid instability by preventing early expression leakage; and protein volumetric productivity of 0.40 g.L^-1^.h^-1^. Alternative induction strategies were also investigated. A mixture of lactose and glycerol as supplementary medium fully induced a high biomass population, reaching a good balance between specific protein production (0.148 g_prot_.g_DCW_^-1^) and volumetric productivity (0.32 g.L^-1^.h^-1^). The auto-induction protocol showed excellent results on specific protein production (0.158 g_prot_.g_DCW_^-1^) in simple batch cultivations. An automated feed control based on the on-line estimated growth rate was implemented, which allowed cells to grow at higher rates than those generally used to avoid metabolic overflow, without leading to acetate accumulation. Some of the protocols described here may provide a useful alternative to standard cultivation and recombinant protein production processes, depending on the performance index that is expected to be optimized. The protocols using glycerol as carbon source and induction by lactose feeding, or glycerol plus glucose in batch medium and induction by lactose pulse led to rSpaA production in the range of 6 g.L^-1^, in short fed-batch processes (16 to 20 h) with low accumulation of undesired side metabolites.

## Introduction

Swine erysipelas causes great economic losses in swine culture worldwide, and has been controlled by the use of live attenuated or inactivated vaccines (Wood 1992). These formulations offer good levels of protection but can aggravate arthritic problems (Freeman 1964; Wood 1992). The surface protein A (SpaA) from *Erysipelothrix rhusiopathiae* is the most studied antigen from this pathogenic bacteria and has shown promising results as a candidate to be used in a subunit vaccine against swine erysipelas (Imada et al. 1999; Kitajima et al. 2000), replacing the cellular vaccines in use.

The production of recombinant antigenic proteins for subunit formulations is among the technologies proposed for the next generation of vaccines (Ashtekar et al. 2012). In case of bacterial antigens, prokaryotic expression systems, such as the *E*. *coli* bacterium, are the most popular choice due the low costs of production, high productivities of heterologous protein and availability of many genetic tools (Demain and Vaishnav 2009).

Recombinant protein production in *E*. *coli* has been extensively investigated, particularly as high cell density cultivations (HCDC) employing bioreactors operating in fed-batch mode and using defined medium (Shiloach and Fass 2005; Choi et al. 2006; Shojaosadati et al. 2008). HCDC studies usually focus on strategies to achieve high biomass concentrations, whereas the induction is triggered by applying simple techniques, such as addition of IPTG pulse (Babaeipour et al. 2007; Carvalho et al. 2011; Khalilzadeh et al. 2004). Thus, induction strategy, feeding control and media formulation are among the main process conditions eligible to be further improved. Besides that, many reports point out for the need to study case by case all these process variables in order to identify the best operation conditions to produce a specific protein.

Induction conditions for proteins under the *lac* promoter control have been largely studied but, in spite of that, just a few new alternative induction strategies have been proposed, such as the so called “auto-induction” (Studier 2005) and the “continuous lactose induction strategy” (Horta et al. 2012; Lim et al. 2004). The auto-induction medium contains glucose, which encourages fast cellular growth in the early stages of the culture, while also inhibiting expression leakage by catabolic repression. Lactose is also present, and is consumed after glucose depletion, activating the lac promoter to induce recombinant protein expression. Glycerol is included to (together with lactose) provide sustained growth during the induction phase (Studier 2005). In spite of the widespread use of auto-induction in shaker flask experiments, only few bioreactor cultures carried out with auto-induction media in batch (Giomarelli et al. 2006; Blommel and Fox 2007a, b) or fed-batch mode (Jia et al. 2011; Xu et al. 2012) have been reported so far. In these studies, bioreactor media contain basically the same carbon and nitrogen sources, but the total carbon source concentration was increased by factors of approximately two (Blommel et al. 2007a, b), three (Giomarelli et al. 2006) or four (Xu et al. 2012) in comparison to the sum of glucose, glycerol and lactose concentrations present in the original auto-induction ZYM-5052 complex formulation (Studier 2005). Consequently, maximum optical densities (600 nm) in the range of ~ 20 to 40 (Giomarelli et al. 2006; Blommel et al. 2007a, b; Jia et al. 2011) were reported for batch experiments and of 140 for fed-batch cultures (Xu et al. 2012). Nevertheless, none of these studies aimed to compare induction strategies. They were focused on biomass production for further purification studies (Giomarelli et al. 2006; Blommel et al. 2007b), on the role of yeast extract or peptone components as inducers or on the influence of aeration on the auto-induction process (Blommel et al. 2007a).

Concerning media formulation, the majority of reports rely on defined media using glucose or glycerol as carbon sources (Shiloach and Fass 2005).The formulation proposed by Neidhardt et al. (1974) containing only simple inorganic salts and a defined carbon source (generally glucose), and improved by the addition of citric acid, EDTA and thiamine-HCl (Riesenberg et al. 1990) is commonly used for *E*. *coli* fed-batch cultures. Korz et al. (1995) used this medium and showed that higher cell densities could be achieved replacing glucose by glycerol. The present work shows that balanced mixtures of both carbon sources can also be used to speed up growth with negligible acetate accumulation. Complex media formulations with glycerol as the main carbon source can also be employed with excellent results.

Regarding the control of supplementary feed flow rate in the fed-batch phase, the conventional strategy manipulates the input of nutrients so that the specific growth rate would not exceed a pre-selected, heuristic, critical value (μ_critic_), to prevent toxic by-products formation. Since glycerol did not trigger acetate accumulation under aerobic conditions (Pinsach et al. 2008), an innovative approach was developed by Horta et al. (2012) to control the carbon source supply in HCDC carried out in defined medium with glycerol. In this work, the exponential feeding flow was tuned using actual rates estimated on-line during the cultivation by a capacitance probe, assuming that the feed of supplementary medium should be limited by the actual growth rates, which reflect the dynamics of the cell metabolic state.

Protein synthesis is also influenced by the chosen cultivation conditions. Usually, defined media and limited growth rates are employed during induction phase (Korz et al. 1995; Lee 1996), which may hamper protein production. However, when cells are in a rich nutritional condition they increase their ribosome content in order to grow faster. Indeed, a linear correlation between cellular ribosome content and growth rate was reported elsewhere (Keener and Nomura 1999). Complex medium and a high growth rate (not limited by a pre-set value during fed-batch operation) probably favor the increase of ribosomal content, which may also improve the productivity of the desired recombinant biomolecule.

In summary, this work presents studies of different cultivation and induction strategies for the production of a recombinant fragment (42 kDa) of the N-portion of SpaA, whose antigenic potential has already been demonstrated in swine vaccination tests (Imada et al. 1999). Gene fragment coding for rSpaA was cloned under the *lacUV5* promoter (Silva et al. 2012). The performance of the conventional bioreactor operation protocol, comprising high biomass accumulation prior to induction of expression by a pulse of lactose or IPTG, was compared with alternative protocols using real-time updated exponential feeding profiles of two supplementary media: a glycerol-based complex medium containing lactose, and the auto-induction medium.

## Materials and methods

Experiments were carried out with *E*. *coli* BL21(DE3) cells producing a recombinant fragment of the SpaA protein from *Erysipelothrix rhusiopathiae*. Cloning procedures were done according to standard protocols (Sambrook and Russell 2001), as previously described (Silva et al. 2012). Briefly, the 1026 pb fragment codifying for the antigenic region of the SpaA protein (Imada et al. 1999) was amplified by PCR from the *E*. *rhusiopathiae* chromosomal DNA and cloned into pGEM-T vector (Promega). After sequencing, the *spa*A fragment was cloned into the expression vector pET28a (Novagen) and used to transform *E*. *coli* BL21(DE3) cells.

Four bioreactor cultivations were conducted under conditions described as follows. Media compositions are presented in Table 1 and Table 2.

**Table 1:** **Media composition used for bioreactor cultivations**

	Experiment #1		Experiment #2		Experiment #3		Experiment #4		
Nutrient (g.L^-1^)	Batch	Fed-batch	Batch	Fed-batch	Batch	Fed-batch	Batch	Fed-batch	Induction
Glucose	10	240.0	8.0	---	---	---	10.0	---	---
Glycerol	---	---	10.0	400.0	40.0	400.0	60.0	400.0	---
Lactose	---	---	2.0	160.0	---	80.0	---	---	20^a^
Tryptone	10.0	10.0	10.0	10.0	10.0	10.0	10.0	10.0	10.0
Yeast extract	5.0	5.0	5.0	5.0	5.0	5.0	5.0	5.0	5.0
NaCl	5.0	5.0	---	---	---	---	---	---	---
MgSO_4_.7H_2_O	2.5	20.0	0.5	40.0	0.5	40.0	0.5	40.0	0.5
K_2_HPO_4_	7.0	7.0	---	---	---	---	---	---	---
KH_2_PO_4_	---	---	3.4	3.4	3.4	3.4	3.4	3.4	3.4
Na_2_HPO_4_.12H_2_O	---	---	9.0	9.0	9.0	9.0	9.0	9.0	9.0
NH_4_Cl	---	---	2.7	2.7	2.7	2.7	2.7	2.7	2.7
Na_2_SO_4_	---	---	0.7	0.7	0.7	0.7	0.7	0.7	0.7
kanamycin (μg.mL^-1^)	100	100	100	100	100	100	100	100	100
Antifoam PPG 30% (mL.L^-1^)	1.0	1.0	1.0	1.0	1.0	1.0	1.0	1.0	1.0
Metal solution	no	no	yes	yes	yes	yes	yes	yes	yes

^a^final concentration in the bioreactor.

**Table 2:** **Metal solution composition**

Component	Batch and induction medium (mg.L^-1^)	Fed-batch medium (mg.L^-1^)
Ferric citrate	100.8	40.0
CoCl_2_.6H_2_O	2.5	4.0
MnCl_2_.4H_2_O	15.0	23.5
CuCl_2_.2H_2_O	1.5	2.3
H_3_BO_3_	3.0	4.7
Na_2_MoO_4_.2H_2_O	2.1	4.0
Zn (CH_3_COO)_2_.H_2_O	33.8	16.0
EDTA	14.1	13.0

Experiment #1: the conventional process strategy was used in this cultivation. A modified LB medium containing glucose as carbon source was used in both batch and fed-batch phases. An exponential feeding profile was set by Eqs. 1 and 2, assuming a constant μ_SET_ = μ_crit_. Induction was carried out by adding a pulse of IPTG, for a final concentration of 0.25 mM in the medium. Two hours after induction, the feeding profile was changed to a constant flow rate, in order to avoid substrate accumulation.

Experiment #2: the auto-induction strategy was employed in this cultivation. Here, induction occurs naturally after glucose exhaustion, as the cells start consuming the lactose present in the medium. The supplementary medium, containing glycerol and lactose as carbon sources was supplied at an exponential feeding flow rate (Eqs. 1 and 2), assuming a constant μ_SET_ = μ_crit_.

Experiment #3: Glycerol was used as sole carbon source during the batch phase. Induction was done along with feed supply, using a feeding medium containing glycerol plus lactose. By this protocol, heterologous protein expression was induced gradually as the supplementary medium was added to the bioreactor. An exponential feeding flow rate was employed based on the actual growth rates of the bacterium to reset both feed control parameters (C_1_ and C_2_ in Equation 1).

Experiment #4: the batch medium was prepared using a combination of glycerol and glucose. Fed batch was carried out exclusively with glycerol as carbon source, using an exponential feeding, again based on the actual growth rates as implemented at Experiment #3 (C_1_ and C_2_ automatically update in Eqs. 1 and 2). Induction was done by a single addition of 400 mL of induction medium containing lactose, after a high biomass concentration was reached.

Inocula were prepared picking up cells from a glycerol stock suspension. The cells were aseptically transferred to the culture medium. Overnight incubation in a rotary shaker was done at 37°C, 250 rpm using the medium of the batch phase of each experiment, with a volume corresponding to 10% of the bioreactor cultivation volume. For the auto-induction strategy (Experiment #2) the inoculum medium was prepared with 0.5 g.L^-1^ glucose and 5.0 g.L^-1^ glycerol, but without lactose.

Experiments were carried out in a 6.0 L bioreactor (*in*-*house made*), connected to a gas analyzer (Sick/Maihak S710). Two mass flow controllers (GFC Aalborg, USA) were employed to supply air and/or oxygen, keeping dissolved O_2_ concentration at 30% of saturation (O_2_ sensor Mettler Toledo, model Inpro 6800) by changing the agitation speed (between 100 and 900 rpm) and enriching air with oxygen when the maximum agitation was reached. The total gas flow rate was restricted between 1 and 2 VVM. The pH was controlled at 7.0 ± 0.2 by adding NH_4_OH 25% (v/v) and H_3_PO_4_ 10% (v/v). All experiments were conducted at 37 ± 0.5°C (growth and induction phases) by controlling the temperature of the water re-circulating in bioreactor jacket. A capacitance probe (Biomass System - Fogale Nanotech) was used for on-line monitoring of biomass formation. The permittivity data collected from the capacitance probe was smoothed by the classical moving average filter (Eilers 2003). The filter used had a size vector of 80 rough data. Online data were used to estimate cell growth rate. The supervisory system SuperSys_HCDC^R^ (Horta et al. 2011Horta et al. 2012), based on LabView (National Instruments), was used to monitor and control the experimental apparatus.

The exponential feeding flow rate for all experiments was calculated by equation 1, which assumes that the specific growth rate depends only on the limiting substrate (in this case, the carbon sources) concentration (Nielsen et al. 2002). The supervisory system controlled the feed, using a peristaltic pump (Ismatec - model BVP).12

*Cx*_*0*_ and *V*_*0*_ are cellular concentration and volume, respectively, which were updated at every 10 min, *Y*_*X*/*S*_ is the biomass yield, *m* the maintenance coefficient (both assumed equal to 0.6 g_DCW_.g_substrate_^-1^ and 0.025 h^-1^, respectively), while *Cs*_*0*_ is the carbon source concentration in the supplementary medium (Table 1), and *Cs*_*R*_ the expected residual concentration of the carbon source in the reactor (assumed as 5 g.L^-1^). When more than one sugar was present in the medium, lactose and/or glucose and/or glycerol were pooled together as one limiting substrate. In Experiments #1-2, the tuning parameters C_1_ and C_2_ were kept constant during all the growth phase, with C_2_ = μ_SET_ = 0.13 h^-1^ (the “critical” specific growth rate, μ_crit_) in Equation 1 and μ= μ_SET_ = 0.13 h^-1^ in Equation 2 (the “classical” approach).

In Experiments #3 and #4, both C_1_ and C_2_ were continuously retuned at each 10 min using the values of μ obtained online from the permittivity measurements provided by the biomass sensor (Horta et al. 2011). Thus, for Experiment #3 and #4, Equations 1 and 2 are no more based on a phenomenological reasoning that imposes a fixed value for μ. Instead, Equations 1 and 2 must be faced as empirical, open loop control laws.

### Analytical methods

Cellular growth: culture broth optical density reading (OD, λ = 600 nm), dry cell weight measurements (g_DCW_.L^-1^), counting CFU.mL^-1^ and on-line estimation by measuring broth permittivity (pF.cm^-1^).

Metabolites and carbon source concentrations were analyzed by HPLC as previously described (Silva et al. 2008). Glucose and glycerol were also monitored throughout the fermentation by a glucose oxidase reaction kit (Laborlab) and a triglycerides determination kit (Laborlab), respectively. Protein expression was checked by SDS-PAGE (Laemmli 1970) and the gels were photographed to estimate the protein production using the software ImageJ (Abramoff et al. 2004). BSA samples with known concentration (0.1, 0.2, 0.4, 0.8 and 1.0 mg.mL^-1^) were used as standards to estimate rSpaA concentration on gels.

To assess plasmid loss during the cultivations, diluted samples of culture broth were initially spread on LB agar plates and incubated for 16 h at 37°C. Seventy five colonies from each sample were simultaneously transferred using sterile toothpicks to new plates with and without kanamycin (30 μg.mL^-1^). After incubation, colonies grown at both conditions for each sample were counted for comparison.

## Results and discussion

### Substrate uptake, biomass formation and metabolite accumulation

**Figure 1:**
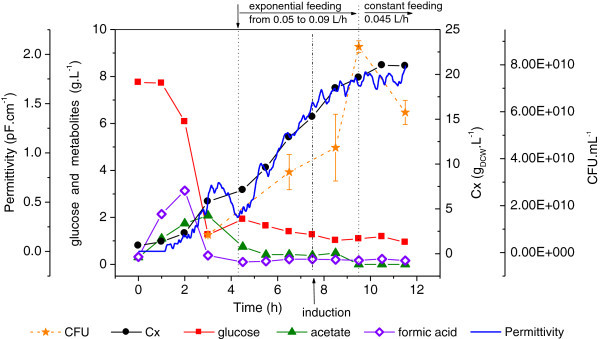
**Cellular growth, substrate consumption and metabolites formation during Experiment #1.** Error bars in CFU are standard deviations from triplicates. Batch and fed-batch using glucose as main carbon source, and IPTG induction.

Experiment#1 was carried out following a classical strategy: fast and intense cellular growth on glucose as sole carbon source (modified LB medium) during batch and feeding phases, and induction with IPTG (0.25 mM final concentration) at the end of the exponential growth, for a period of 4 hours. A maximum biomass concentration of 22 g_DCW_.L^-1^ was achieved at the end of the cultivation. The controlled addition of supplementary feed, together with good aeration conditions, prevented accumulation of by-products at the end of the process (Figure 1). Acetic and formic acids formed during the batch phase were consumed in the period between glucose exhaustion (after 3 h) and feeding start up (beginning at 4.2 h), and were not present in significant amounts (maximum concentration below 0.5 g.L^-1^) during the induction phase. Permittivity data followed closely the trends of biomass formation, but with drastic slope changes that marked the beginning and end of cell growth on acetate. It should be noticed, however, that the decrease in permittivity between 3.5 and 4.5 h (i.e., a strong decrease in viable cell mass) was a trend not followed by the measurements of DCW and OD.

The capacitance probe measures the dielectric permittivity of cell suspensions, which is related not only to biomass concentration, but also to the cell structure as well as to its morphological and physiological states (Matanguihan et al. 1994). A relationship between capacitance and cell viability has also been suggested (Matanguihan et al. 1994; Asami 2002).

A maximum specific growth rate of 1.1 h^-1^ was registered, even in the presence of acetic and formic acids. Biomass yield coefficient was around 0.6 g_DCW_.g_glucose_^-1^ during the batch phase (Table 3).

**Table 3:** **Comparison of cell growth results, including standard deviations of estimated kinetic parameters**

	Experiment #1	Experiment #2	Experiment #3	Experiment #4
μ_max_ (h^-1^)	1.12 ± 0.05	0.80 ± 0.27	0.62 ± 0.05	1.08 ± 0.02
Y_x/s_ (batch phase)	0.60 ± 0.01	0.57 ± 0.04	0.59 ± 0.05	0.64 ± 0.04
Final Cx (g.L^-1^)	20.9	13.5	41.9	66.1
[acetate] maximum (g.L^-1^)	2.1	5.0	3.7	1.2
[formate] maximum (g.L^-1^)	3.1	N.D.	N.D.	N.D.

N.D.: not detected; μ_max_ refers to the maximum observed specific growth rate.

**Figure 2:**
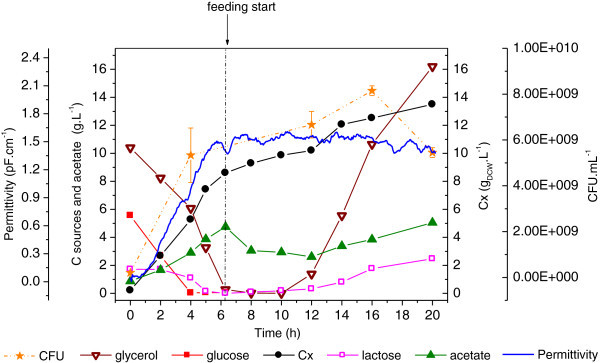
**Cellular growth, substrate consumption and metabolites formation during Experiment #2.** Auto-induction protocol using glucose, glycerol and lactose as carbon sources in batch phase. Feeding stage with glycerol and lactose. Error bars in CFU are standard deviations from triplicates.

The auto-induction strategy was tested in Experiment #2. As shown in Figure 2, consumed glucose in batch phase resulted in biomass accumulation in a short period of time. Glycerol and lactose were assimilated simultaneously, resulting in glycerol accumulation from the mid fed-batch phase on, due to the high glycerol and lactose concentrations present in the supplementary feed. There was no formic acid formation as observed in the cultivation with glucose as the sole carbon source (Experiment #1). But, acetate formation was observed throughout the cultivation. Together with metabolic stress associated to protein over expression, acetate probably affected cellular viability.

Maximum specific growth rate was 0.80 h^-1^, and biomass yield coefficient was 0.57 g_DCW_.g_substrate_^-1^, both a little lower than with glucose (Experiment #1), as expected (Table 3).

Permittivity data detected the drop in cellular viability from the mid fed-batch phase on, as also shown by CFU counting. Such a reduction cannot be observed in the dry cell weight data as this method does not distinguish between viable and non-viable cells.

From the growth profile depicted at Figure 2, it seems that the anticipated expression overloaded cellular metabolism and affected cell division, despite the abundance of nutrients. It caused glycerol accumulation and low biomass formation. However, lactose remained intensely consumed until the end of cultivation.

Glycerol was used as sole carbon source in the batch medium for Experiment #3. An alternative induction strategy was also studied here, where the feeding medium was prepared with a combination of glycerol and lactose to promote rSpaA expression and sustain growth at the same time.

As it has been reported in the literature, *E*. *coli* cells grow slower on glycerol than on glucose due to differences in the substrate assimilation mechanisms (Korz et al. 1995; Lee 1996). But, on the other hand, growth on glycerol leads to lower toxic byproducts formation as this lower assimilation rate does not cause metabolic overflows (Lee 1996; Tripathi et al. 2008).

**Figure 3:**
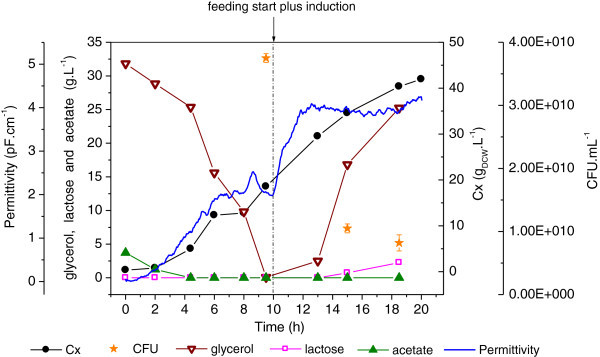
**Cellular growth, substrate consumption and metabolites formation during Experiment #3.** Growth on glycerol during batch phase; fed-batch and induction using glycerol plus lactose. Error bars in CFU are standard deviations from triplicates.

As shown in Figure 3, permittivity data followed quite well the trends of biomass formation during the first 10 h of cultivation. However, when the fed-batch and induction phase started, a bias in the biomass concentration profiles estimated by the capacitance probe with respect to DCW is observed. This could be attributed to the increased metabolic activity during protein expression, captured by the capacitance probe. CFU measurements at ~15 h of cultivation (5 h of induction) showed an opposite trend in comparison to both DCW experimental points and permittivity data. Here a discussion of how to assess cell density in the absence of other techniques such fluorescence flow cytometry and real time PCR arises. CFU accounts for viability of cells taken off the bioreactor environment, DCW may be biased by non-viable cells and capacitance may be affected by changes in the dielectric properties of cells where a great accumulation of heterologous protein occurs.

As also observed in Experiment #2, glycerol accumulation occurred after the mid fed-batch phase but lactose was almost completely consumed, showing that glycerol concentration can be reduced in the formulation of the supplementary medium. In this experiment, the growth deceleration during heterologous protein expression was not as intense as in Experiment #2, neither was the accumulation of lactose. Therefore, lower lactose concentration– for instance, in the range of 80 g.L^-1^ (half of that used in Experiment #2) – might be enough to promote an efficient induction in a gradual manner. The absence of lactose in the batch medium and the control of μ matching the actual growth rate were other successful changes implemented in this experiment. Together, they allowed intense growth towards 40 g_DCW_.L^-1^ within 20 h of cultivation. As discussed by Sandén et al. (2002), higher specific growth rates favor protein production by influencing both the protein synthesis apparatus and the energy production.

**Figure 4:**
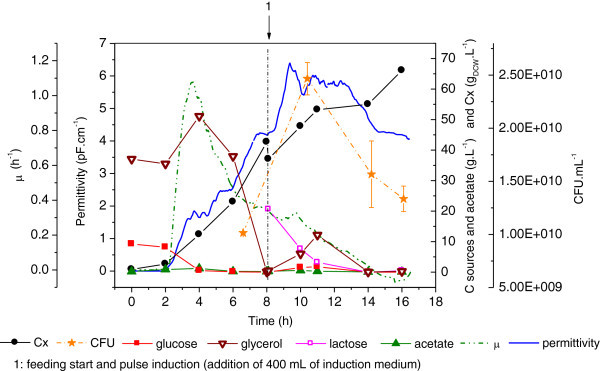
**Cellular growth, substrate consumption and metabolites formation during Experiment #4.** Growth on glycerol and glucose during batch phase; Fed-batch using glycerol and induction by a lactose pulse. Error bars in CFU are standard deviations from triplicates.

Experiment #4 was carried out according to the conventional induction strategy, using a pulse of inducer after biomass is accumulated, but with lactose instead of IPTG to induce recombinant protein synthesis. Results of this cultivation are shown in Figure 4.

Biomass formation was improved by a combination of glucose and glycerol in the batch phase and after only 7 h a cellular concentration around 30 g. L^-1^ was reached. Despite such an intense cellular growth, there was no accumulation of organic acids.

Permittivity data matched the biomass formation profile up to 8 h from the onset of the experiment, when induction, by a lactose pulse, took place. As also observed in Experiment #3, as protein expression triggers an increase of metabolic activity, the capacitance probe signal is affected (see Figure 4, between 8–10 h).

A maximum of 66 g_DCW_.L^-1^ was achieved after only 16 h of cultivation. Such a high biomass productivity can be associated mainly to two aspects of this experiment: i) the improved batch medium, containing glycerol plus glucose and; ii) the feeding rate controlled in such a way to sustain the real growth rate of the cells, which was inferred on-line from data collected by the capacitance probe. In fact, at the beginning of the exponential feeding phase (6.8 h of cultivation), the value of μ_SET_ was around 0.35 h^-1^, which is significantly higher than the pre-defined μ_SET_ used in Experiments #1 and 2 (0.13 h^-1^).

The free software SuperSys_HCDC^R^ (Horta et al. 2011,2012) was used to control the feeding flow rate. Based on the broth permittivity signal from the capacitance probe, the supervisory system retuned the parameters of the feed flow control each 10 min. Thus, biomass growth was limited by the cells own growing capacity, and not by the parsimonious feed of a limiting substrate. The feeding flow decreased with time after induction, accompanying the growth deceleration caused by the recombinant protein expression.

Glycerol as carbon source in the supplementary medium did trigger the aerobic acetic fermentation as reported by Korz et al. (1995), but the production of acetate was negligible. This is a remarkable difference with respect to growing cells on glucose at high rates. The saturation of the cellular catabolic fluxes, typical for cultivations using glucose, is not observed for glycerol, due to its lower uptake rate by the cells. This fact allows one to feed the bioreactor in such a way that cells are able to grow at high specific rates without accumulation of toxic by-products. On the other hand, during the batch phase, a balanced mixture of both carbon sources leads to fast biomass formation but also without acetic acid accumulation.

During the fed-batch phase, lactose consumption led to a slight accumulation of glycerol, as lactose, besides its intrinsic induction activity, acts as a carbon source. However, after lactose depletion, the accumulated glycerol was readily consumed.

A comparison of results from the four cultivations is presented in Table 3.

As it can be seen in Table 3, the highest μ_max_ value was achieved when cells were cultivated using glucose (Experiment #1); cultivation #3, where glycerol was the carbon source, showed the lower maximum specific growth rate, as expected. Intermediate values of μ_max_ were observed where a mixture of substrates was used (#2 and #4). The balanced composition used in Experiment #4 led to a high μ_max_ value, of 1.08 h^-1^. Table 3 also shows that biomass yield coefficients were not significantly influenced by the carbon sources present in the rich media. As expected, growing on glucose (#1) led to significant amounts of formic and acetic acids production, while in the other experiments no formic acid nor other toxic metabolic by-products were detected at all. Lactose feeding in Experiments #2 and #3 induced acetate production, which is related to the degradation of this sugar into glucose and galactose. The latter is not consumed by *E*. *coli* BL21(DE3) cells, but glucose metabolism could favor acetate formation under the stressing conditions of heterologous protein over expression.

#### Induction strategy and protein production

The rSpaA protein was expressed in the form of inclusion bodies and different levels of protein production were reached according to the induction strategy adopted. For the classical process, where cells were grown in modified LB medium and induced by an IPTG pulse (Experiment #1), the maximum rSpaA specific production reached 0.14 g_prot_.g_DCW_^-1^ after four hours of induction. This value corresponds to approximately 25% of total cellular protein content – considering that total cellular protein content is around 55% of its dry weight (Neidhardt and Umbarger 1999).

**Figure 5:**
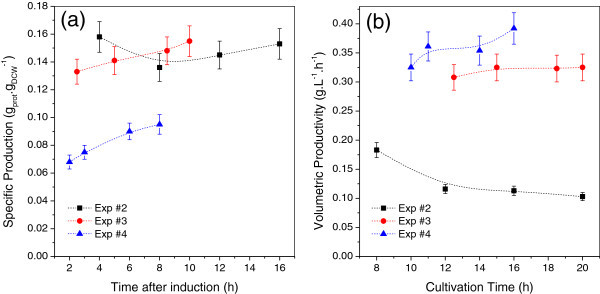
**Comparison of specific rSpaA production (a) and volumetric productivity (b) over time for the lactose induced protein expression.** For Experiment #2, the induction was assumed to start after 4h from the onset of the experiment, which corresponds to the moment when glucose was exhausted.

In spite of that, IPTG is expensive, toxic and must be completely removed from the final product to comply with GMP guidelines. To replace the use of IPTG, other induction strategies were tested using lactose as inducer, and the progression of protein production along time was evaluated for these experiments (Figure 5a, b).

A great efficiency for recombinant protein production was registered for the auto-induction protocol (Experiment #2). Lactose from batch and feeding media gradually activated rSpaA expression and the highest specific protein production from all experiments performed (0.16 g_prot_.g_DCW_^-1^) was achieved after 8 h of culture. On the other hand, the high metabolic load, caused by prolonged and intense induction, impaired cellular growth.

The auto-induction protocol showed to be very efficient to achieve high levels of protein expression within a short period. However, it does not seem to be appropriated for fed-batch cultures because the high cellular concentrations desired in this mode of operation cannot be reached due to the intense metabolic burden associated to the prolonged and early induction process.

Feeding lactose as inducer in the supplementary medium (Experiment #3) resulted in high rSpaA specific and volumetric productivity at the end of the cultivation (0.15 g_prot_.g_DCW_^-1^ and 0.323 g_prot_.L^-1^.h^-1^, respectively), which confirm the good performance of the alternative induction protocol. Recombinant SpaA accumulated up to almost 6.0 g_prot_.L^-1^, corresponding to around 27% of total cellular protein content after 8.5 h of induction.

Addition of a lactose pulse to induce recombinant protein expression was tested in Cultivation #4. Although the rSpaA specific production was the lowest registered in all the four experiments, the volumetric productivity reached was the highest among all the strategies, due to the great biomass productivity.

The lactose concentration of 20 g.L^-1^ in the moment of induction was superior to values commonly reported in the literature (Li et al. 2006; Neubauer et al. 1992). It led to an amount of 76.5 g of lactose totally consumed within 3.3 h after its addition. Compared to the total amount of lactose fed in the other experiments in the same 3.3 h (10.5 g in #2 and 36.1 g in #3), the value of 76.5 g used in #4 was not too low to sustain the same pattern of induction in the continuously growing cell population.

Once biomass concentration during induction phase of Experiment #4 was significantly higher than in Experiments #2 and #3, a better comparison is provided by estimating the overall specific lactose uptake rates (OSLUR) and overall specific glycerol uptake rates (OSGUR) for each of them during the first 3.3 h of induction. Rather similar values for OSLUR (0.09 for Experiments #2 and #3 and 0.11 g_lactose._g_DCW_^-1^ h^-1^ for Experiment #4) and for OSGUR (0.47 and 0.41 g_glycerol._g_DCW_^-1^ h^-1^ for Experiments #3 and # 4, respectively) were observed. The OSGUR of Experiment #2 was rather lower (~ 0.23 g_glycerol._g_DCW_^-1^ h^-1^) due to the reduced glycerol uptake associated to the low biomass build-up caused by the early protein expression triggered by auto-induction protocol. Thereby, these results suggest that the inferior specific protein production observed at Experiment #4 was not caused by lack of lactose. Instead, it seems to be related mainly to the way lactose was supplied. It could be associated to the modulation of the transcription rate provided by the continuous inducer feeding (Exps #2 and #3), which led to extended production phase and better yields (Striedner et al. 2003). The same differences between pulse induction and continuous inducer dosage using IPTG were reported elsewhere (Pinsach et al. 2008), and better results were also achieved using the inducer feeding protocol.

**Figure 6:**
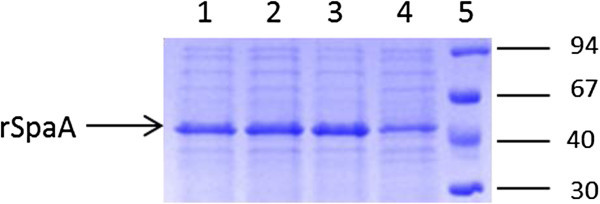
**SDS-PAGE of cell lysates from samples collected during bioreactor cultures of*E*.*coli*BL21(DE3) expressing rSpaA under different culture and induction conditions (Exp #1 to #4).** Lane 1: Exp #1 (t = 11.5 h); Lane 2: Exp #2 (t = 8 h); Lane 3: Exp #3 (t = 20 h); Lane 4: Exp #4 (t = 16 h); Lane 5: Molecular mass markers (kDa). Samples were collected at the cultivation time (t) indicated in parentheses, that corresponds to the moment of maximum specific protein production for each experiment.

As shown in Figure 6, the maximum specific protein production was similar for experiments #1, #2 and #3, and higher than for Experiment #4. In spite of that, as stated before, the highest volumetric productivity was reached at Experiment #4, since rSpaA production did not impair cellular growth and high biomass concentration was reached in a short period of time. Subsequent lactose pulses could be done to increase the specific protein production. Similarly, the addition of the supplementary feed containing lactose could be delayed and preceded by a glycerol containing feed medium to improve biomass accumulation.

For the auto-induction experiment (#2), the reasonable values of specific protein production and volumetric productivity reached after only 8 h of cultivation (that corresponds to a few hours after the end of the batch phase) shows that such protocol is better suited for short experiments, namely batch cultures, where high protein production can be achieved within a few hours by the early induction of expression.

The protein production profile observed in the auto-induction, fed-batch culture carried out in the present study (Figure 5) is different from the continuously increasing protein production profile attained for the auto-induction, fed-batch cultures of Xu et al. (2012). These authors used higher glucose concentration (30 g/L) and lower inducer concentration (~0.2 g/L) in the batch medium. In addition, a lower temperature (28°C) and a constant rate of feeding supply were adopted. So, protein production during batch phase was not expressive, and during the fed-batch phase the induction of protein synthesis took place at slower rate, relieving the metabolic burden and resulting in the increasing profile, even after 18 h of culture. On the other hand, such a strategy impairs productivity, which is an important performance index to be optimized.

In the other experiments, with shorter periods of induction, specific protein production and volumetric productivity did not decrease when the growth rate decelerated. Experiment #1, induced by IPTG, had the shortest induction phase and specific protein production and volumetric productivity were evaluated only at the end of the process (0.136 g_prot_.g_DCW_^-1^ and 0.25 g.L^-1^.h^-1^, respectively).

In fact, Figure 5 shows that time plays a less important role than expected during the induction phase. First of all, data depicted at Figure 5a, b show that significant protein accumulation in the cell was already attained after 2 – 3 h of continuous induction with lactose, what is rather shorter than the usual, minimum 8 h of induction. Generally speaking, the specific production (protein/cell mass) increased slightly with time (with one exception at the beginning of induction of Experiment #2) while the effect on volumetric productivity of the protein was not monotonic, due to the different growth rates after induction. Experiment #4, in particular, showed a trend towards increasing productivities as time goes by.

Table 4 compares results from this work to other recent published data for *E*. *coli* cultures employed for biopharmaceutical proteins production. The strategy used in Experiment #4 was able to achieve a protein volumetric productivity among the highest reported, after a short bioreactor run (16 h). It should be noticed that the recombinant biomolecule produced here has 42 kDa, while the others are much smaller proteins, being in the range of 7 to 28 kDa, except for streptokinase.

**Table 4:** **Comparative performance for production of some biopharmaceutical proteins by*E*.*coli*BL21(DE3) fed-batch cultures in complex medium**

Product & molecular weight	Carbon source/inducer	Culture time (h)	Biomass productivity (g_DCW_.L^-1^.h^-1^)	Protein volumetric productivity (g_prot_. L^-1^.h^-1^)	Specific protein production (g_prot_.g_DCW_^-1^)	Product content (g.L^-1^)	Reference
Human soluble B lymphocyte stimulator (hsBLyS) 17-18 kDa	Glucose/lactose	34	0.92	0.11	0.120	3.70	Li et al. 2006
Tilapia insulin-like growth factor 2 - 7 kDa	Glucose/IPTG	30	5.20	0.32	0.062	9.69	Hu et al. 2004
Streptokinase 47 kDa	Glucose/IPTG	11	1.31	0.10	0.050	1.12	Goyal et al. 2009
Anti-cancer drug TATm-survivin (T34A) 17 kDa	Glucose/IPTG	14	0.63	0.12	0.191	1.68	Zhang et al. 2009 (t = 14 h)
Anti-HIV protein Griffthsin (GRFT) 14.5 kDa	Auto induction (Glucose + glycerol + lactose)/lactose	15	0.89^a^	0.05	0.061^a^	0.82	Giomarelli et al. 2006
**this work:**							
SpaA from *E*. *rhusiopathiae* 42 kDa	Glucose/IPTG	11.5	1.82	0.25	0.136	2.85	Exp #1 (t = 11.5 h)
	Auto induction (Glucose + glycerol + lactose)/lactose	20	1.16	0.18	0.158	1.47	Exp #2 (t = 8 h)
	Glicerol/lactose	20	2.18	0.32	0.155	5.97	Exp #3 (t = 20 h)
	Glycerol + glucose/lactose	16	4.13	0.39	0.095	6.35	Exp #4 (t = 16 h)

^a^estimated values, assuming 1 unit of Optical Density at 600 nm (OD_600nm_ = 1.0) equivalent to 0.5 g_DCW_.

“t” corresponds to the cultivation time where the maximum values for specific protein production were reached (for each experiment).

Both specific protein production and volumetric productivity impact on process performance. In addition, for bioprocess optimization, the economics of each solution must be assessed, including the cost for disruption of cells and separation and purification of the protein.

Cellular productivity reflected the induction strategy and media formulation that were adopted. Conditions with prolonged periods of induction (Experiments #2 and #3) led to lower biomass productivity caused by the intense stress imposed to the cells. Formulations with glucose (#1) or glucose plus glycerol (#4) promoted fast growth, reducing the total time of the process.

Plasmid maintenance is also strongly influenced by the induction strategy employed. It remained between 96 and 100% before induction for all experiments, and decreased in different intensities according to the induction regimen adopted, ranging from 70% (Exps #2 and #3) to 95% (Exp #4) at end of the processes. The metabolic burden imposed to plasmid-carrying cells after induction causes a growth deceleration, which favors the growth of plasmid-free cells, generated by unequal partition of plasmids between daughter cells (Summers 1991). Shorter periods of induction may prevent the accumulation of plasmid-free cells, avoiding a reduction in the protein productivity with time. Glucose in the batch medium is also supposed to have a beneficial effect on plasmid maintenance, as it represses expression leakage before induction by catabolite repression (Zhang et al. 2003).

## Conclusions

The production of heterologous proteins under the control of *lac* promoter in r*E*.*coli* fed-batch cultures has been widely studied in the last 15 years. But, the majority of the reported studies focus on IPTG as inducer and defined media containing glucose as major carbon source. Here we showed that lactose supplied continuously as part of feeding medium formulation is an effective approach to fully induce protein expression.

Concerning carbon sources, the balanced combination of glucose and glycerol in the batch medium is a simple way to boost growth even with glycerol as main substrate. Furthermore, during the fed-batch phase, growth on glycerol can be kept as high as 0.35 h^-1^ in complex medium without metabolite formation. This enables faster biomass production leading to shorter cultivations. Even when glycerol accumulated to 30 g.L^-1^, neither metabolite formation nor inhibition of protein synthesis occurred, as it would actually happen if glucose was used, instead.

The auto-induction protocol also studied here showed to be best suited for simple batch cultivations, where great amounts of recombinant protein can be reached in a short period of time by running an easy-to-do experiment.
